# Lifestyle and mental health 1 year into COVID-19

**DOI:** 10.1038/s41598-021-02702-4

**Published:** 2021-12-02

**Authors:** Paolo Nicola Barbieri, Osea Giuntella, Silvia Saccardo, Sally Sadoff

**Affiliations:** 1 Centre for Health Economics, University of Gothenburg, Gothneburg, Sweden; 2 Department of Economics, University of Pittsburgh, Pittsburgh, USA; 3 Institute of Labor Economics - IZA, Bonn, Germany; 4 Department of Social and Decision Sciences, Carnegie Mellon University, Pittsburgh, USA; 5 University of California San Diego, San Diego, USA

**Keywords:** Human behaviour, Risk factors

## Abstract

In previous work, Giuntella et al. (Proc Natl Acad Sci 118:e2016632118, 2021), we documented large disruptions to physical activity, sleep, time use and mental health among young adults at the onset of the COVID-19 pandemic in Spring 2020. This study explores the trends 1 year into COVID-19, as vaccines began to roll out, COVID-19 deaths declined, and social distancing measures eased in the United States. We combine biometric and survey data from multiple cohorts of college students spanning Spring 2019 through Spring 2021 (N = 1179). Our results show persistent impacts of the pandemic on physical activity and mental health. One year into the pandemic, daily steps averaged about 6300 per day compared to about 9800 per day prior to the pandemic, a 35% decline. Almost half of participants were at risk of clinical depression compared to a little over one-third prior to the pandemic, a 36% increase. The impacts on screen time, social interactions and sleep duration at the onset of COVID-19 largely dissipated over the course of the pandemic, though screen time remained significantly higher than pre-pandemic levels. In contrast to the sharp changes in lifestyle and mental health documented as the pandemic emerged in March 2020, we do not find evidence of behavioral changes or improvements in mental well-being over the course of Spring 2021 as the pandemic eased.

## Introduction

The COVID-19 pandemic led to unprecedented disruptions to nearly every facet of daily life. At the onset of the public health emergency, lockdowns and social-distancing measures suddenly altered the way people interacted, worked and attended school, breaking old habits and shaping new ones. In^1^, we leveraged a unique dataset linking biometric and survey data among young adults and documented large disruptions in physical activity, sleep, social interactions and screen time at the onset of the pandemic in Spring 2020. These lifestyle changes arose alongside large declines in mental health, with a significant increase in the proportion of young adults at risk of clinical depression.

In the current study, we extend the dataset in^1^ to examine the *persistence* of lifestyle and mental health disruptions 1 year into the pandemic. We address two questions: (1) Were the disruptions documented at the onset of the pandemic transitory, with people restoring their pre-pandemic habits after an initial period of adaptation? And (2) To what extent have people returned to their pre-pandemic habits as vaccines rolled out, COVID-19 deaths declined, and social distancing measures eased?

Our study contributes to a large literature on habit formation and adaptation to environmental changes. The psychological literature reports little long-term sensitivity of subjective well-being to environmental changes, as individuals have been found to adapt to both adverse and beneficial life circumstances^2,3^. Behavioral research on exercising documents limited persistence to newly induced habits through policy intervention^4,5^, suggesting that pandemic-induced changes in physical activity may be temporary. On the other hand, habits are vulnerable to disruptions of the environmental cues that trigger a given behavior^6^, and the COVID-19 pandemic has generated dramatic and sudden changes to everyday circumstances. Survey evidence from July-October 2020 finds that US adults expect longer term changes in their post-COVID work, travel, and exercise habits, including more walking and biking post-pandemic^7^.

Understanding the persistence of the pandemic’s impacts on lifestyle and mental well-being is critical for informing policy^8^. estimated the short term physical and mental health costs of the pandemic in the US to be $2.6 trillion and $1.6 trillion, respectively. The costs could be substantially higher if the impacts persist. This is particularly the case for habits such as exercise, that have longer term health consequences^9^. If lifestyle habits and mental well-being do not naturally rebound as the pandemic eases, then interventions may be required to help people return to normalcy.

### Longitudinal study

We compare five cohorts of college students at the University of Pittsburgh from Spring 2019 through Spring 2021 (N = 1179). The median age in our sample is 19 and 95% of the sample is under 23. Each cohort participated in a semester-long study that collected biometric data via wearable devices (Fitbits) along with survey measures of time use and mental well being. The first two waves occurred in Spring 2019 and Fall 2019, prior to the pandemic. The Spring 2020 cohort began participating before the pandemic in February 2020 and continued through the onset of the pandemic in March and April 2020, with a subset extending past the end of the semester into July 2020. The Fall 2020 wave took place from September-November 2020 during the rise of cases in the second wave of COVID-19 in the US. Our final cohort participated in Spring 2021 from February to May, which spans the period in the US when vaccines started becoming more widely available, COVID-19 deaths rapidly declined and social distancing measures eased. Vaccines became available to adults (over 16) statewide on April 19, 2020. By the end of May, 95% of the students in our sample had received at least the first-dose and 85% had received both doses.

We consider the onset of the pandemic as beginning March 23, 2020—when University classes moved online after an extended spring break (March 9–22, 2020)—through the end of the term (April 20, 2020). Instruction resumed in person in the Fall of 2020, while the University maintained limitations to gatherings throughout the 2020–2021 academic year.

### Lifestyle disruptions

**Figure 1:**
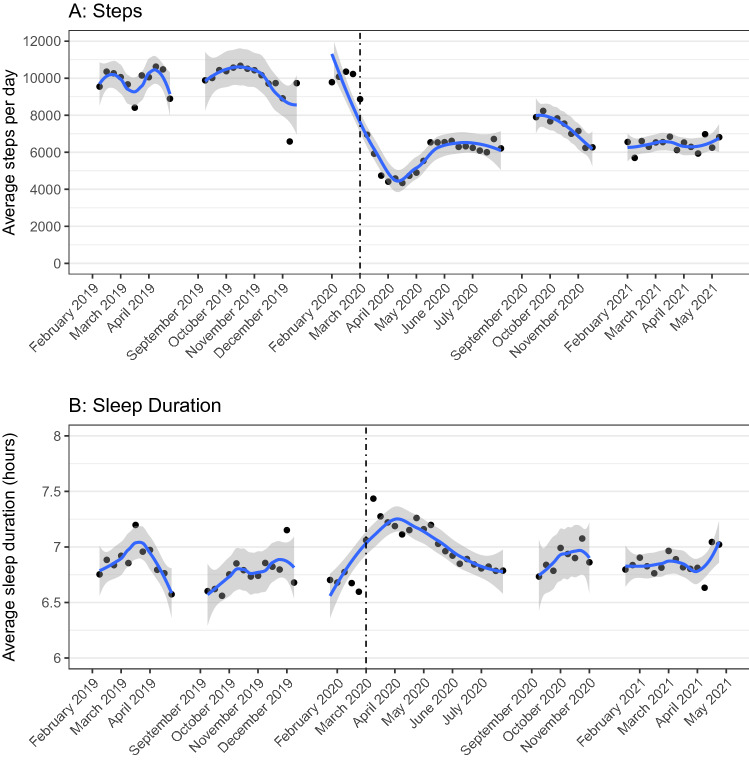
(**A**,**B**) The figure plots the average outcomes by week for study participants in the Spring 2019 through Spring 2021 cohorts for all participants with Fitbit data (n = 1150). Gray shading indicates 95% confidence intervals for the locally weighted smoothing curve. This figure was produced using R (version 3.6.1) https://www.r-project.org/.

Figure 1 plots average daily physical activity and sleep across the semester for each cohort for all individuals with Fitbit data (n = 1150). Prior to the pandemic, daily steps averaged about 9800 steps per day across cohorts (Fig. 1A). In March 2020, steps sharply declined to about 4,600 steps per day through the end of the term. After the semester ended, steps rebounded slightly to 6300 steps per day in May–July 2020. Average daily steps increased slightly in Fall 2020 to 6900 per day. In Spring 2021, steps returned to Spring 2020 levels averaging about 6,400 per day. We find a similar pattern for daily active (non-sedentary) time, which averaged about 4.3 h prior to the pandemic, dropped to 2.9 h per day at the onset of the pandemic, rebounded in May 2020 to 3.6 h per day and remained at 3.6 and 3.7 h per day in Fall 2020 and Spring 2021, respectively. All pre- versus post-pandemic differences for steps and physical activity are significant at the $p<0.001$ level (see SI for discussion of estimation of differences). Sleep duration (Fig. 1B), which increased by about 30 min at the onset of the pandemic ($p<0.001$ from a differences in differences regression compared to Spring 2019), largely returned to pre-pandemic levels by June 2020 through Spring 2021, averaging around 7 h per night in Spring 2021 ($p=0.91$ compared to Spring 2019).

**Figure 2:**
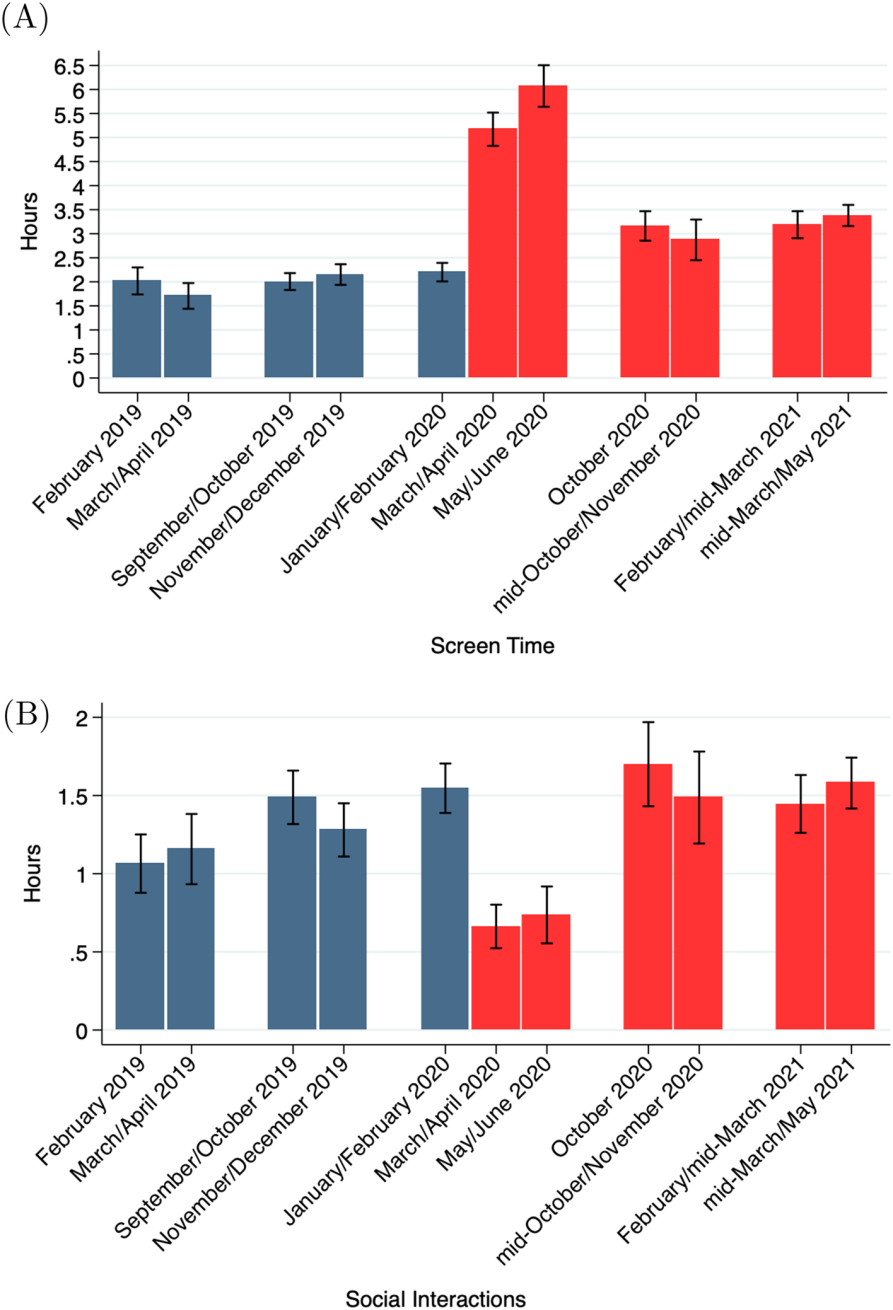
Screen time and social interactions. The figures show the average screen time and the average time spent with friends (social time) during the Spring 2019 through Spring 2021 terms for all participants with time use data (N = 1122). Screen time includes time spent playing games, watching television, or surfing the Internet and does not include time spent working or studying on a device. Bars indicates 95% confidence intervals. This figure was created using Stata (version 14.1) http://www.stata.com.

Figure 2 displays self-reported time use at the beginning and end of each semester for all individuals with time use data (N = 1122). Panel A shows average screen time, which includes time spent playing games, watching television or surfing the internet and does not include time spent working or studying on a device. Self-reported screen time more than doubled at the onset of the pandemic in Spring 2020 from about 2.2 h per day at the start of the term to 5.2 h per day at the end of the term ($p<0.001$). However, by Fall 2020 screen time had substantially declined ($p<0.001$ compared to the onset of the pandemic) and remained steady through Spring 2021, averaging about 3.2 h per day in the 2020–2021 academic year, still significantly higher than pre-pandemic levels ($p<0.001$).

Self-reported social interactions (Fig. 2B), which declined by over half from 1.5 h to about 40 min per day at the onset of the pandemic ($p<0.001$), fully recovered by Fall 2020, averaging about 1.5 h per day in the 2020–2021 academic year ($p=0.081$ compared to pre-pandemic levels). The decline in screen time and restoration of time spent with friends occurred during the Fall 2020 term while COVID-19 cases were still high. There were no further significant changes over the course of Spring 2021 as the vaccine became available to all adults and cases declined ($p=0.11$ for screen time, $p=0.17$ for social interactions comparing Spring 2021 baseline and endline).

### Mental health

**Figure 3:**
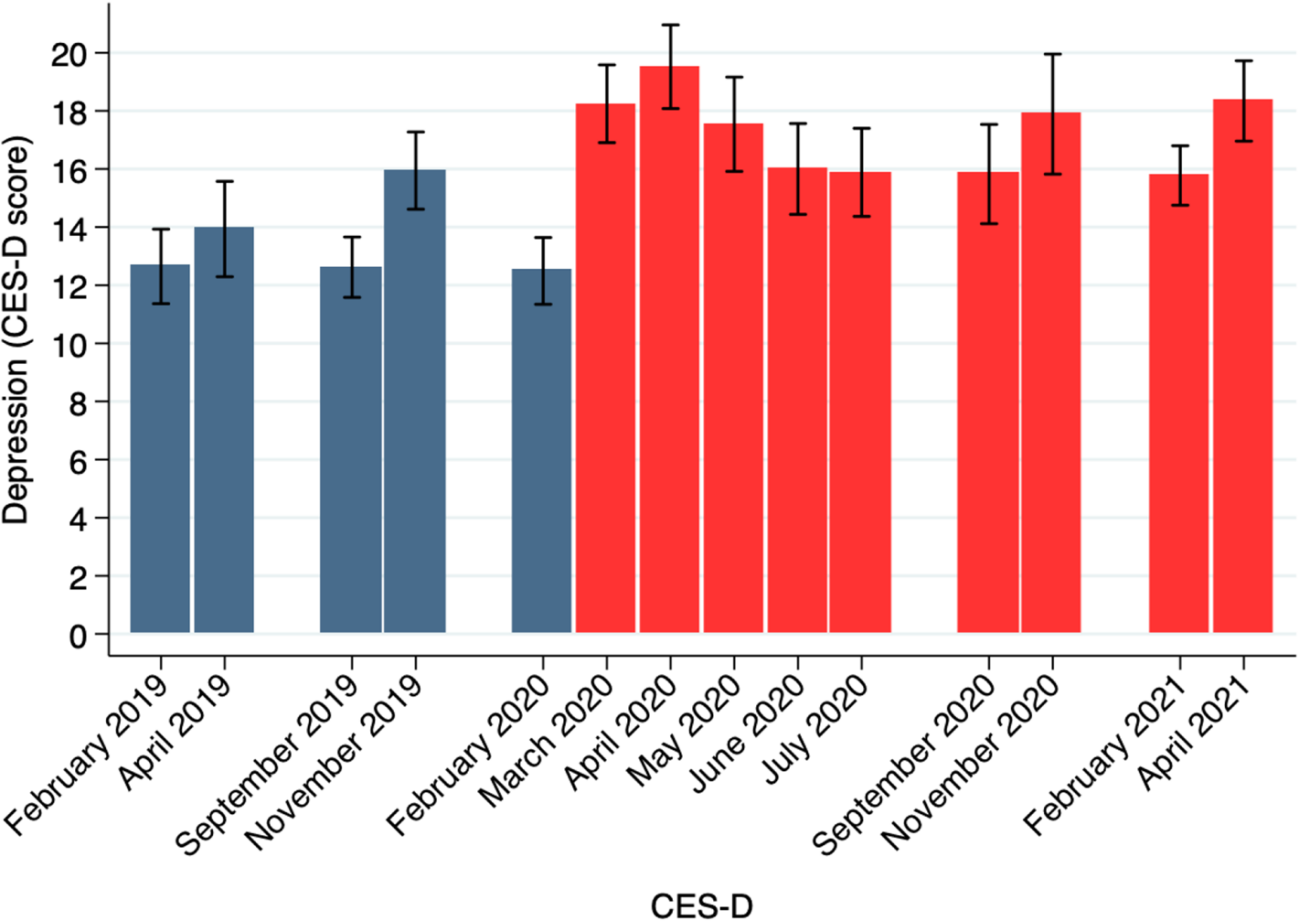
Depression. The figure show the average Center for Epidemiological Depression (CES-D) scale scores for the Spring 2019 through Spring 2021 terms (n = 1179). Bars indicates 95% confidence intervals. This figure was created using Stata (version 14.1) http://www.stata.com.

Our primary measure of mental health, the Center for Epidemiological Depression (CES-D) scale^10^, shows large increases in symptoms of depression experienced in Spring 2020 that largely persist through Spring 2021 (Fig. 3, n = 1179). We report the full list of depression symptoms used in the CES-D scale in the SI Appendix (Table S7). At the onset of the pandemic, average CES-D scores increased by about 50% in a single month between February (baseline) and March (midline) 2020 from 12.4 to 18.2, reaching a peak of 19.5 in April 2020 ($p<0.001$ for all differences compared to baseline). Average scores declined in May 2020 ($p=0.005$ compared to the onset of the pandemic) and remained between 15.8–18.3 through Spring 2021, averaging 16.8 in the 2020–2021 academic year, 24% higher than pre-pandemic levels of 13.6 ($p<0.001$).

Using a CES-D score of 16 or higher as the cutoff for critical concern^11^, we estimate that a year into the pandemic about 42-56% of our participants were at risk for clinical depression. Average rates were 47% in Spring 2021 compared to about 35% of participants prior to the pandemic, a 34% increase ($p<0.001$). Similar to physical activity, sleep and time use, we see no evidence of improvements in mental well-being over the Spring 2021 semester, with CES-D scores increasing as they generally do in our dataset between the beginning and the end of the term ($p<0.001$ comparing Spring 2021 baseline and endline). Our results are consistent with^12,13^, which review studies of mental health over the course of the pandemic. They find large declines in mental health at the onset of the pandemic with recovery in some indices of well-being, but less so for measures of depression. Here we show that the mental health decline persists among young adults, a particularly vulnerable population^14^.

### Conclusion

In this paper, we show that some of the initial disruptions to lifestyle and mental health documented at the onset of the pandemic persisted throughout Spring 2021. Notably, these effects are stable over the course of Spring 2021, when vaccines started becoming more widely available, COVID-19 related deaths dropped, and society began to ease restrictions. As society moves back towards normalcy, researchers, policy makers and public health experts should keep in mind these long-lasting effects of the pandemic, which threaten long-term physical and mental health. Developing interventions to reduce sedentary habits and improve well-being will be crucial to protect the physical and mental health of young adults.

## Methods

We enrolled five cohorts of students from the University of Pittsburgh in a semester-long wellness study (AEA RCT ID AEARCTR- 0003235): Spring 2019 (N = 150), Fall 2019 (N = 315), Spring 2020 (N = 300), Fall 2020 (N = 131), Spring 2021 (N = 366). The study includes N = 1262 participant-term observations from N = 1179 unique participants (n = 83 participated in both Fall 2019 and Spring 2020). The study was approved by the University of Pittsburgh Institutional Review Board and all methods were performed in accordance with the relevant guidelines. All informed consent was obtained from all subjects and/or their legal guardian(s). Data and materials can be accessed at Open Science Framework (https://osf.io/39gk6/).

All participants received a Fitbit (Alta HR or Inspire), which allowed us to track their biometric data (daily steps, physical activity, and sleep) throughout the semester. Participants filled out an enrollment survey that collected baseline information and measured mental health using the Center for Epidemiologic Studies Depression (CES-D) scale^10^, which was repeated at the end of the semester. In 2020, we also collected data in mid-March right after the closure of campus facilities, in May a month after the end of the term, in June, and in July. The CES-D score is a measure of self-reported well-being designed to assess the frequency of symptoms of depression (e.g., loneliness, feeling depressed, loss of appetite, feeling sad) on a scale from 0 (rarely or none of the time) to 3 (most or all of the time) and has a total score between 0 and 60. Throughout the semester, we collected weekly measures of time use using a diary survey following the structure of the American Time Use survey^15^.

We estimate differences during the pandemic compared to the same period prior to the pandemic using OLS regressions and clustering the standard errors at the individual level (see Tables S2–S7 in SI Appendix). Formally,

1 $$\begin{aligned} y_{it}=\beta Period_{it}+\delta + X_{it}+\epsilon _{it} \end{aligned}$$

where $y_{it}$ is the outcome of interest for participant *i* in period *t* and $\beta$ is the coefficient for the term ($Period_{it}$) that is being compared to the same period prior to the pandemic. For instance, if referring to Spring 2021 versus the relevant pre-pandemic period (e.g., Spring 2019), we restrict the sample to observations of the relevant period in Spring 2019 and compare them with the observations of Spring 2021. If we are referring to averages in the 2020–2021 academic year, we would compare the pooled pre-pandemic observations (Spring 2019, Fall 2019 and Spring 2020 baseline) to the Fall 2020 and Spring 2021 cohorts. $X_{it}$ are individual characteristics including age, gender, race, financial aid, and self-reported health at baseline. Tables S2–S7 report estimates with and without including these controls.

For the analysis of differences at the onset of the pandemic we use a differences-in-differences estimation, as in^1^. We use the following regression:

2 $$\begin{aligned} y_{it}=\beta Endline*y2020+\gamma Endline+ \lambda y2020 +\delta X_{it} + \epsilon _{it} \end{aligned}$$

where *Endline* is a dummy for endline vs baseline of a term and *y*2020 is a dummy for whether an individual was observed in 2020. All the *p*-values reported in the paper are obtained using regressions as above (see Tables S2–S7). The codes are available in the Web Appendix at this url https://osf.io/39gk6/. The analysis includes all observations for the relevant outcome. We note that our sample restrictions differ slightly from those in^1^. A detailed description of the methods and measures can be found in SI Appendix.

## Supplementary Information

Supplementary Information.
