# Author Correction: Lifestyle and mental health 1 year into COVID-19

**DOI:** 10.1038/s41598-021-03388-4

**Published:** 2021-12-08

**Authors:** Paolo Nicola Barbieri, Osea Giuntella, Silvia Saccardo, Sally Sadoff

**Affiliations:** 1 Centre for Health Economics, University of Gothenburg, Gothneburg, Sweden; 2 Department of Economics, University of Pittsburgh, Pittsburgh, USA; 3 Institute of Labor Economics - IZA, Bonn, Germany; 4 Department of Social and Decision Sciences, Carnegie Mellon University, Pittsburgh, USA; 5 University of California San Diego, San Diego, USA

Correction to: *Scientific Reports* 10.1038/s41598-021-02702-4, published online 02 December 2021

The original version of this Article contained errors. The Funding section was incomplete.

"Open access funding provided by University of Gothenburg."

now reads:

“The project was funded by J-PAL North America. Open access funding provided by University of Gothenburg.”

Additionally, Figure 2 contained an error in the top of panel (A), where erroneous text was added.

**Figure 2:**
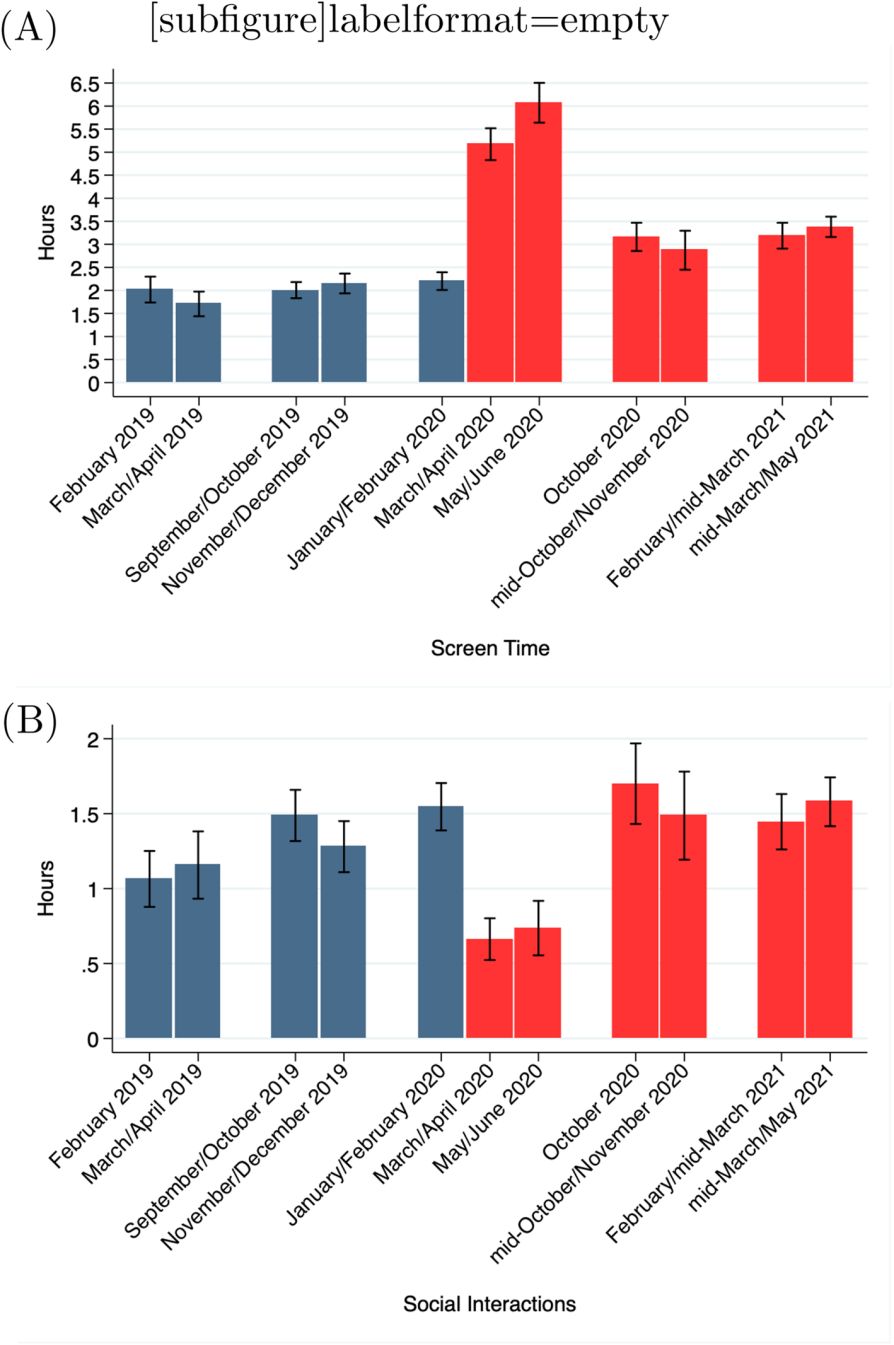
Screen time and social interactions. The figures show the average screen time and the average time spent with friends (social time) during the Spring 2019 through Spring 2021 terms for all participants with time use data (N = 1122). Screen time includes time spent playing games, watching television, or surfing the Internet and does not include time spent working or studying on a device. Bars indicates 95% confidence intervals. This figure was created using Stata (version 14.1) http://www.stata.com.

The original Figure 2 and its accompanying legend appear below.

The original Article has been corrected.

