# Supplementary material for: Lifestyle and mental health 1 year into COVID-19
Source: Sci Rep. 2021 Dec 2;11:23349. doi: 10.1038/s41598-021-02702-4 (PMC8640003; doi:10.1038/s41598-021-02702-4)
Supplement: Supplementary file 1 — Supplementary Information. [file 41598_2021_2702_MOESM1_ESM.pdf]

Supplementary Information for
Lifestyle and mental health one year into COVID-19

Paolo Nicola Barbieri*, Osea Giuntella, Silvia Saccardo and Sally Sadoff

Supplementary Text

1 Data, Setting and Recruitment

In February 2019, we began a study which enrolled participants in a semester-long randomized experiment on wellness (AEA RCT ID AEARCTR- 0003235). As part of the study, we collected a rich longitudinal data set of measures of students' physical health (physical activity, sleep, heart rate), time use, and mental health. The study was approved by the University of Pittsburgh Institutional Review Board. We have collected data from five cohorts of students in Spring 2019, Fall 2019, Spring 2020, Fall 2020, and Spring 2021. For a full description of the randomized trial, see [1].

At the beginning of each semester, we invited college students at the University of Pittsburgh to enroll in a semester long experiment on wellness. We recruited participants from the Pittsburgh Experimental Economics Lab using the SONA online management system. To be eligible for the study, participants had to have a smartphone and be willing to wear and routinely synchronize a wearable device (Fitbit) throughout the semester. Students interested in participating in the study received $6 for taking part in an initial 30-minute session in the laboratory (this initial session was held online in Fall 2020 and Spring 2021) and received an additional payment at study completion. Participants were guaranteed a payment of $30 for the study, and the opportunity to receive additional earnings based on luck and their decisions in the study. During the initial session of the study, each participant received a Fitbit device (Alta HR or Inspire), registered for a Fitbit account, and installed a custom-made smartphone app on their smartphone, which allowed us to track their

*Corresponding author: paolo.barbieri@economics.gu.se

Fitbit data and to interact with the subjects throughout the study. In Fall 2020 and Spring 2021, we mailed the Fitbit devices to participants. During the initial session, we instructed participants to wear their device as much as possible, synchronize it daily, answer weekly surveys, and return the device at the end of the semester. All participants filled out an enrollment survey where we collected baseline information on demographics, self-reported health, and mental health.

Throughout the semester, students filled out either weekly or bi-weekly surveys about time use, and received weekly text messages alernately measuring mood and resilience using experience sampling techniques. At the end of the study, they filled out an endline survey which contained most of the questions we collected at baseline, including the mental health questions.

In the Spring 2020 cohort, we also administered a midline survey on March 20th, right after the university had announced that classes would be moved online and students were encouraged not to come back to campus. The survey assessed the frequency of social interactions (face to face and via text-messages, calls and video calls), changes in day-to-day behaviors as a result of the pandemic, changes in employment status and living conditions, participants' beliefs over the pandemic, and mental health. We additionally collected information on individual location after the University announced remote learning.

After a baseline period, we assigned participants to interventions to improve their sleep for four weeks (intervention period, see the pre-registration, AEA RCT ID AEARCTR- 0003235) and then continued to follow them in the post-intervention period.

At the end of the study, participants filled out an endline survey repeating most of the measures collected at the beginning of the semester, including mental health. Then, participants received any outstanding payments they had earned through surveys or other decisions they made in the study. If their cumulative payments over the course of the study were less than $30, we added to their payment the amount required to make their total payment equal $30, as that was the guaranteed minimum cumulative earnings indicated in recruitment materials.

In Spring 2019, we followed participants from February through the end of the semester (end of April), in Fall 2019, we followed participants from mid-September to early December. In Spring 2020, the wellness study began in February and was set to conclude at the end of the semester (April 20th). We obtained approval from the University of Pittsburgh Institutional Review Board to invite participants to continue their participation in a follow up study. At the end of the Spring 2020 term in April, we gave participants the option to stay enrolled in the study through the summer. A total of 205 participants elected to stay enrolled and signed the consent form. We continued following these participants through May, June and July. Throughout this period we sent reminders to sync the Fitbit and administered time use and experience sampling surveys every week, as we did during the semester. In Fall 2020,

we followed participants from the end of September 2020 until the until the third week of November 2020, as classes started and finished earlier because of COVID-19 mitigation strategies. In Spring 2021, we followed subjects from mid-February until the end of May.

Our sample consists of N=150 participants in Spring 2019, N=315 participants in Fall 2019, N=300 participants in Spring 2020, N=131 participants in Fall 2020 and N=366 participants in Spring 2021. This sample includes N=83 people who enrolled in the study in Fall 2019 and continued participating through Spring 2020. The total sample includes N=1262 participant-term observations from N=1179 unique participants. 96% of our sample is comprised by students aged 18-23. The remaining 4% includes older students with only 1% of them being over 30 years old.

2 Main Variables

2.1 Mental Health

All mental health questions were collected in the baseline (upon enrollment) and endline survey. There were N=1179 surveys filled out at baseline, when participants enrolled in the study in the various terms. Of these, 79% filled out the endline survey (N=133 in Spring 2019, N=284 in Fall 2019, N=211 in Spring 2020, N=115 in Fall 2020, N=238 in Spring 2021). For Spring 2020, we also have a midline survey, which was filled out by 83.1% of participants (N=195). We additionally measured mental health in May, June and July 2020 for the subset of participants who continued the study after the semester ended (N=167, N=174 and N=171, respectively). The pre-pandemic period includes the Spring 2019 cohort, the Fall 2019 cohort and Spring 2020 baseline. A copy of the surveys can be found on online at https://osf.io/f85e3/.

Depression. To measure depression, we administered the Center for Epidemiologic Studies Depression Scale-D scale (CES-D, [2]). The CES-D scale is a widely used validated self-report instrument designed to assess depressive symptomatology in the general population. The scale is comprised of 20 items that assess the frequency of symptoms associated with depression, such as feelings of sadness, loss of appetite, or loneliness, using a scale from 0 (Rarely or None of the Time) to 3 (Most or all of the times). An overall depression score is obtained by summing answers to all 20 questions (see Table S1), with higher scores indicating greater depressive symptoms. Individuals with scores at or above the threshold of 16 points [3] are classified to be at risk of clinical depression. We did not record any information on suicide and no adverse events were reported throughoiut the study.

2.2 Physical Activity and Sleep

Participants were given devices (Fitbit ¢ Alta HR,Fitbit ¢ Inc, San Francisco, CA). Personal wearable activity trackers, such as Fitbits, have been used in past work to study health behavior [1]. Through the wearable trackers we

collected data on physical activity and sleep. In particular, we collected data on daily steps and heart rate. We also collected data on sleep duration, wake up time, bedtime, and sleep disruptions. Because the devices measure heart rate, they are able to distinguish between time spent in bed not sleeping, such as watching TV, and time spent sleeping. The devices combine measures of heart rate and movement to measure daily active (non-sedentary) time, which is measured as minutes in which a person is nonsedentary for at least 10 continuous minutes, where nonsedentary minutes are defined as activity that raises heart rate enough to burn at least 1.5 times as many calories as at rest. In practice, this measure sums the lightly active, fairly active and very active minutes collected by the Fitbit. Resting heart rate is the number of heart beats per minutes while at rest (the typical resting heart rate is 60-80 beats per minute).

We include observations from N=1150 participants who have Fitbit data (we are missing Fitbit data for N=29 participants who did not successfully sync their Fitbit to the smartphone app collecting the data). The pre-pandemic period includes the Spring 2019 cohort (N=133), the Fall 2019 cohort (N=306) and the Spring 2020 cohort (N=297) through March 11. In regressions and averages reported in the paper, in 2020 we we exclude observations between March 12 to March 22, because students were on spring break, which was extended to two weeks when classes went remote starting on March 23rd. We consider the "onset of the pandemic" as beginning March 23, 2020 and going through the end of the term (April 20, 2020). Including the spring break days or using an alternative cutoff for the "onset of the pandemic" does not qualitatively change the results. We track a subset of participants who continued the study after the Spring 2020 semester ended in May through July 2020 (N=205). Of these, 195 participants have Fitbit data. In June 2020, we conducted a randomized experiment. We exclude from the June-July 2020 steps analysis participants who were randomly assigned to an intervention that incentivized physical activity from June 1-June 14, 2020, leaving us with N=94 non-treated individuals with Fitbit data for those two months. See [1] for details of the intervention. The 2020-21 academic year includes the Fall 2020 cohort (N=129) and the Spring 2021 cohort (N=365).

2.3 Time use

Throughout the study, participants filled out time-use diaries every 2 weeks or every week depending on the term. They indicated how they spent the previous 24 hours by choosing from different categories, following the structure of the American Time Use Survey https://www.bls.gov/tus). We categorize time use into the following categories: social interactions, screen, work, study, eating or preparing food, personal care, sleep, exercise, errands and commuting. Social interactions measure time spent hanging out with friends. Screen time includes time spent watching TV, playing video games or surfing the internet, and excludes study or work time on the screen.

We report average screen time and social interactions for surveys filled out in February 2019 and March/April 2019 for the Spring 2019 cohort; September/October 2019 and November/December 2019 for the Fall 2019 Cohort; January/February 2020, March/April 2020 and May/June 2020 for the Spring 2020 cohort; early October 2020 and mid-October/November 2020 for the Fall 2020 cohort; February/mid-March 2021 and mid-March/May 2021 for the Spring 2021 cohort.

When comparing time use at the beginning and end of the term, we consider baseline periods to include September/October for Fall and January/February for Spring terms; and endline periods to include mid-October/December for Fall and March/April for Spring terms (the baseline period extends into mid-March in Spring 2021). The baseline and endline periods differ across terms in part because the schedule of the academic year changed during the COVID-19 Pandemic. In 2020-21, the academic year the Fall term started and finished a week earlier than in previous years, while the Spring term started a week later than previous years There was also no Spring break in 2021. Results are not substantially affected by the dates used to define baseline and endline periods. With the exception of Spring 2020, there are only marginal differences in either social interactions or screen time within a given term.

A total of 1122 participants (95.1% of our sample) filled out at least one time-use survey during the baseline period (N=132 in Spring 2019, N=298 in Fall 2019, N=244 in Spring 2020, N=131 in Fall 2020, N=334 in Spring 2021). A total of 1060 participants (89.9% of our sample) filled out at least one survey during the endline period (N=121 in Spring 2019, N=265 in Fall 2019, N=209 in Spring 2020, N=113 in Fall 2020, N=352 in Spring 2021). In Spring 2020, we additionally surveyed a subsample who continued after the term ended in May/June 2020 (N=193). The pre-pandemic period includes the Spring 2019 cohort, the Fall 2019 cohort and Spring 2020 baseline.

3 Sample

We include all participants who have a baseline CES-D measure collected during enrollment at the beginning of the semester. In all of our figures and analysis, we include every observation we have for a given outcome measure. Hence, sample sizes differ depending on the outcome measure of interest. Note that this approach differs from [1], where we restricted the figure and some analysis to participants who had an endline CES-D measure at the end of the semester. In this paper, there are N=1179 unique participants, 83 of whom took part in the study in Fall 2019 and continued through Spring 2020. Of these, N=1150 synced the Fitbit allowing us to collect biometric data. Further, a total of 1078 unique participants filled out at least one time use survey.

4 Analysis

We estimate differences during the pandemic compared to the same period prior to the pandemic using OLS regressions and clustering the standard errors at the individual level. Formally,

Formally,

$$y_{it} = \beta Period_{it} + \delta + X_{it} + \epsilon_{it} \tag{1}$$

where y_{it} is the outcome of interest for participant i in period t and β is the coefficient for the term ($Period_{it}$) that is being compared to the same period prior to the pandemic. For instance, if referring to Spring 2021 vs. the relevant pre-pandemic period (e.g., Spring 2019), we restrict the sample to observations of the relevant period in Spring 2019 and compare them with the observations of Spring 2021. If we are referring to averages in the 2020-21 academic year, we would compare the pooled pre-pandemic observations (Spring 2019, Fall 2019 and Spring 2020 baseline) to the Fall 2020 and Spring 2021 cohorts. X_{it} are individual characteristics including age, gender, race, financial aid, and self-reported health at baseline. Tables S2-S7 report estimates with and without including these controls.

For the analysis of differences at the onset of the pandemic we use a differences-in-differences estimation, as in [1]. We use the following regression:

$$y_{it} = \beta Endline * y2020 + \gamma Endline + \lambda y2020 + \delta X_{it} + \epsilon_{it} \tag{2}$$

where $Endline$ is a dummy for endline vs baseline of a term and $y2020$ is a dummy for whether an individual was observed in 2020. All the p-values reported in the paper are obtained using regressions as above. The codes are available in the Web Appendix at this url https://osf.io/39gk6/.

Table S1 CES-D components

1. I was bothered by things that usually don't bother me.	11. My sleep was restless.
2. I did not feel like eating; my appetite was poor.	12. I was happy.
3. I felt that I could not shake off the blues even with help from my family or friends.	13. I talked less than usual.
4. I felt I was just as good as other people.	14. I felt lonely.
5. I had trouble keeping my mind on what I was doing.	15. People were unfriendly.
6. I felt depressed.	16. I enjoyed life.
7. I felt that everything I did was an effort.	17. I had crying spells.
8. I felt hopeful about the future.	18. I felt sad.
9. I thought my life had been a failure.	19. I felt that people disliked me.
10. I felt fearful.	20. I could not get going.

Table S2 DiD analysis for steps

	Steps													
	(1)	(2)	(3)	(4)	(5)	(6)	(7)	(8)	(9)	(10)	(11)	(12)	(13)	(14)
Mar-Apr 20 - Pre-Covid	-5167.7***	-5106.0***												
	(0.000)	(0.000)												
May-July 20 - Pre-Covid			-3427.8***	-3359.0***										
			(0.000)	(0.000)										
Fall 20 - Pre-Covid					-2882.9***	-3252.4***								
					(0.000)	(0.000)								
Fall 20 - Fall 19							-3024.2***	-3380.2***						
							(0.000)	(0.000)						
Spr 21 - Pre-Covid									-3373.7***	-3325.2***				
									(0.000)	(0.000)				
Spr 21 - Spr 19											-3721.4***	-4457.0***		
											(0.000)	(0.000)		
Sept 20 - May-July 20													2209.8	1597.2
													(0.171)	(0.353)
Observations	44744	44744	43991	43991	46011	46011	28633	28633	47821	41993	12744	6916	9413	9413
R^2	0.122	0.143	0.049	0.072	0.045	0.064	0.068	0.090	0.070	0.056	0.106	0.149	0.001	0.066
Controls	No	Yes	No	Yes	No	Yes	No	Yes	No	Yes	No	Yes	No	Yes
n. of individuals	523	523	523	523	652	652	435	435	865	655	475	265	204	204

p-values in parentheses

Note: Controls include age, gender, race, poor mental health and financial aid.

Standard errors are adjusted for clustering at the individual level

* $p < 0.05$, ** $p < 0.01$, *** $p < 0.001$

Table S3 DiD analysis for physical activity

	Physical Activity									
	(1)	(2)	(3)	(4)	(5)	(6)	(7)	(8)	(9)	(10)
March-April 20 - Pre-Covid	-1.531***	-1.514***								
	(0.000)	(0.000)								
Fall 20 - Pre-Covid			-0.742***	-0.910***						
			(0.000)	(0.000)						
Fall 20 - Fall 19					-0.816***	-0.956***				
					(0.000)	(0.000)				
Spring 21 - Pre-Covid							-0.821***	-0.804***		
							(0.000)	(0.000)		
Spring 21 - Spring 19									-0.803***	-1.131***
									(0.000)	(0.000)
Observations	44744	44744	46011	46011	28633	28633	47821	41993	12744	6916
R^2	0.077	0.103	0.021	0.043	0.036	0.056	0.029	0.040	0.032	0.068
Controls	No	Yes	No	Yes	No	Yes	No	Yes	No	Yes
n. of individuals	523	523	652	652	435	435	865	655	475	265

p-values in parentheses
Note: Controls include age, gender, race, poor mental health and financial aid.
Standard errors are adjusted for clustering at the individual level
* $p < 0.05$, ** $p < 0.01$, *** $p < 0.001$

Table S4 DiD analysis for sleep

	Sleep					
	(1)	(2)	(3)	(4)	(5)	(6)
Spring 20 - Spring 19	0.361***	0.355***				
	(0.000)	(0.000)				
Spring 21 - Spring 19 (all semester)			-0.00771	0.0518		
			(0.909)	(0.573)		
Spring 21 - March-April 20					0.0468	0.0754
					(0.519)	(0.476)
Observations	8884	8884	37104	17305	11777	6332
R^2	0.011	0.033	0.000	0.026	0.000	0.031
Controls	No	Yes	No	Yes	No	Yes
n. of individuals	367	367	496	276	472	264

p-values in parentheses

Note: Controls include age, gender, race, poor mental health and financial aid.

Standard errors are adjusted for clustering at the individual level

* $p < 0.05$, ** $p < 0.01$, *** $p < 0.001$

Table S5 DiD analysis for screen time

	Screen-Time									
	(1)	(2)	(3)	(4)	(5)	(6)	(7)	(8)	(9)	(10)
Spring 20 (end - base)	2.972***	3.008***								
	(0.000)	(0.000)								
Fall 20 - Pre-Covid			0.973***	1.073***						
			(0.000)	(0.000)						
Fall 20 - March-April 20					-2.146***	-2.310***				
					(0.000)	(0.000)				
Fall20 + Spring 21 - Pre-Covid							1.164***	1.131***		
							(0.000)	(0.000)		
Spring 20 (end - base)									0.192	0.0974
									(0.105)	(0.607)
Observations	453	453	1304	1304	453	453	1990	1569	686	265
R^2	0.343	0.370	0.048	0.084	0.182	0.214	0.082	0.106	0.002	0.047
Controls	No	Yes	No	Yes	No	Yes	No	Yes	No	Yes
n. of individuals	253	253	759	759	339	339	1120	898	361	139

p-values in parentheses

Note: Controls include age, gender, race, poor mental health and financial aid.

Standard errors are adjusted for clustering at the individual level

* $p < 0.05$, ** $p < 0.01$, *** $p < 0.001$

Table S6 DiD analysis for social interactions

	Social Interactions											
	(1)	(2)	(3)	(4)	(5)	(6)	(7)	(8)	(9)	(10)	(11)	(12)
Spring 20 (end - base)	-0.884***	-0.877***										
	(0.000)	(0.000)										
Spring 21 - Pre-Covid			0.156	0.219								
			(0.081)	(0.163)								
Fall 20+Fall 21 - Pre-Covid					0.178*	0.202						
					(0.025)	(0.051)						
Fall 20+Spring 21 - Pre-Covid							0.178*	0.202				
							(0.025)	(0.051)				
Fall 21 - Pre-Covid									0.243	0.176		
									(0.051)	(0.171)		
Spring 21 (end - base)											0.133	0.0129
											(0.171)	(0.934)
Observations	453	453	1746	1325	1990	1569	1990	1569	1304	1304	686	265
R^2	0.128	0.155	0.003	0.050	0.004	0.048	0.004	0.048	0.005	0.051	0.002	0.039
Controls	No	Yes	No	Yes	No	Yes	No	Yes	No	Yes	No	Yes
n. of individuals	253	253	990	768	1120	898	1120	898	759	759	361	139

p-values in parentheses
Note: Controls include age, gender, race, poor mental health and financial aid.
Standard errors are adjusted for clustering at the individual level
* $p < 0.05$, ** $p < 0.01$, *** $p < 0.001$

Table S7 DiD analysis for mental health

	CESD										CESD > 15			
	(1)	(2)	(3)	(4)	(5)	(6)	(7)	(8)	(9)	(10)	(11)	(12)	(13)	(14)
Spring 20 (midline - base)	5.796***	5.784***												
	(0.000)	(0.000)												
Spring 20 (endline - base)			7.070***	6.999***										
			(0.000)	(0.000)										
May 20 end - Spring 20 base					-1.978**	-2.115**								
					(0.005)	(0.002)								
A. year 20-21 - Pre-Covid							3.180***	3.737***						
							(0.000)	(0.000)						
Spring 21 (endline - base)									2.564***	3.195**			0.141***	0.211***
									(0.000)	(0.001)			(0.000)	(0.000)
Spring 21 - Pre-Covid											0.210***	0.390***		
											(0.000)	(0.000)		
Observations	431	431	428	428	378	378	1949	1583	604	238	1337	1194	604	238
R^2	0.089	0.109	0.119	0.157	0.009	0.044	0.024	0.040	0.015	0.054	0.027	0.049	0.019	0.081
Controls	No	Yes	No	Yes	No	Yes	No	Yes	No	Yes	No	Yes	No	Yes
n. of individuals	266	266	270	270	218	218	1179	956	366	143	920	777	366	143

p-values in parentheses

Note: Controls include age, gender, race and financial aid.

Standard errors are adjusted for clustering at the individual level

* $p < 0.05$, ** $p < 0.01$, *** $p < 0.001$

References

[1] Giuntella, O., Hyde, K., Saccardo, S., Sadoff, S.: Lifestyle and mental health disruptions during covid-19. Proceedings of the National Academy of Sciences **118**(9) (2021)

[2] Devin, G., Orme, C., Costello, C., *et al.*: Measuring depressive symptoms in illness populations: psychometric properties of the ces-d scale. Psychol Health **2**(139), 56 (1988)

[3] Radloff, L.S.: The ces-d scale: A self-report depression scale for research in the general population. Applied psychological measurement **1**(3), 385–401 (1977)
